# Supplementary material for: CREBBP inactivation sensitizes B cell acute lymphoblastic leukemia to ferroptotic cell death upon BCL2 inhibition
Source: Nat Commun. 2025 May 20;16:4274. doi: 10.1038/s41467-025-59531-6 (PMC12092839; doi:10.1038/s41467-025-59531-6)

Supplementary Data Table 1: Cell viability data from small molecule screen in 697^{WT}, 697^{KI} and 697^{KO} cell lines (related to figure 1). One-way Brown-Forsythe and Welch ANOVA test with Dunnett T3 comparison comparing to 697^{WT} used for statistical analysis, unless specified with a * where unpaired T-test used. n=3 technical replicates.

Supplementary Data Table 2: Differentially regulated proteins. Two-sided limma statistical test. To control for the false discovery rate (FDR), p-values were adjusted using the Benjamini-Hochberg method for multiple testing correction.

Supplementary Data Table 3: Lipidomics raw data, standards and parameters.

Supplementary Data Table 4: Two-sided Pearson correlation with p-value adjusted for multiple testing using Bonferroni correction of gene expression with *CREBBP* expression in the TARGET phase 2 RNAseq cohort. Gene sets for ferroptosis are shown.

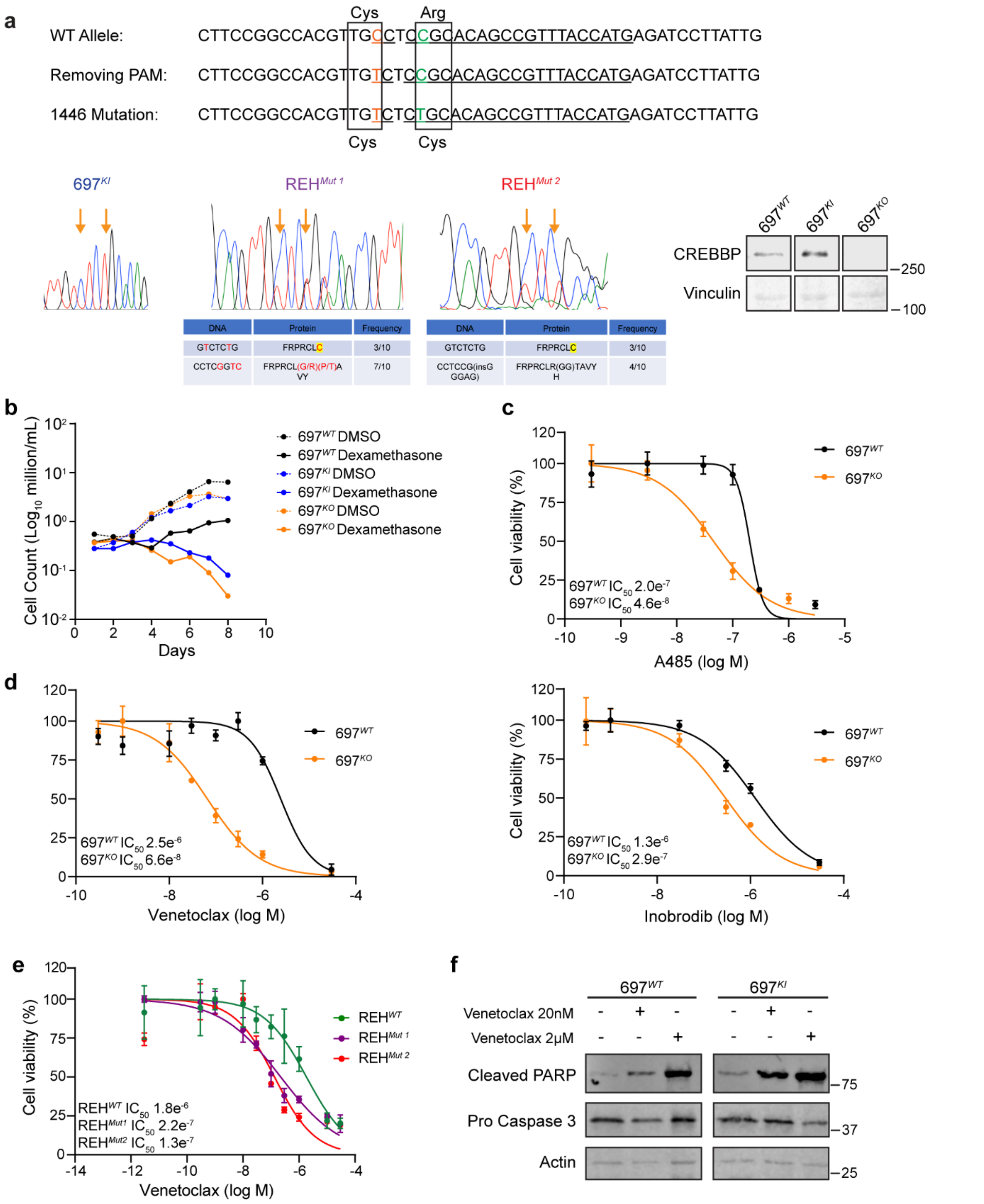

Supplementary Figure 1: *CREBBP*-mutated B-ALL cell lines show increased sensitivity to Venetoclax. a, Summary of genome editing strategy. Top: Two single base substitutions were introduced by CRISPR directed homologous recombination to: i) generate the R1446C mutation; and ii) remove the protospacer adjacent motif (PAM) to prevent further Cas9 binding and repeat cutting of successful edits. Bottom left: Results of amplicon sequencing showing a homozygous edit in 697^{KI} cells and a compound heterozygous edit in two REH mutant clones. The alternative sequences for the remaining two alleles were sequenced on TOPO-TA cloned amplicon fragments (sequences, amino acid substitution and TOPO-TA clonal frequency are shown in table below). Bottom right: Western blot of

CREBBP vs. Vinculin protein in 697 edited clones, confirming loss of protein in 697^{KO} cells. Marker sizes in kDa shown.

b, Growth curves of 697^{WT} (black), 697^{KI} (blue) and 697^{KO} (yellow) grown in the presence of DMSO vehicle (hashed lines) or 10nM Dexamethasone (solid lines). Independent duplicate.

c, Dose response curves of two CREBBP/EP300 inhibitors A485 (top) and Inobrodib (bottom) showing enhanced sensitivity of 697^{KO} (yellow) compared to 697^{WT} (black) in 72h MTS viability assay. n=3 technical replicates, mean±SD.

d, Dose response curve of 697^{WT} (black) and 697^{KO} (yellow) lines to Venetoclax in 72h MTS viability assay. n=3 technical replicates, mean±SD.

e, Dose response curve of REH^{WT} (green) and two isogenic *CREBBP*-mutant clones (purple and red) to Venetoclax in 72h MTS viability assays. n=3 technical replicates, mean±SD.

f, Western blot for cleaved PARP (top) and cleavage of pro-caspase 3 (middle) in 697^{WT} (left) and 697^{KI} (right) cell lines. Cells were incubated with DMSO vehicle or Venetoclax at either 20nM or 2000nM concentrations. Actin is shown as a loading control (bottom).

Source data are provided as a Source Data file.

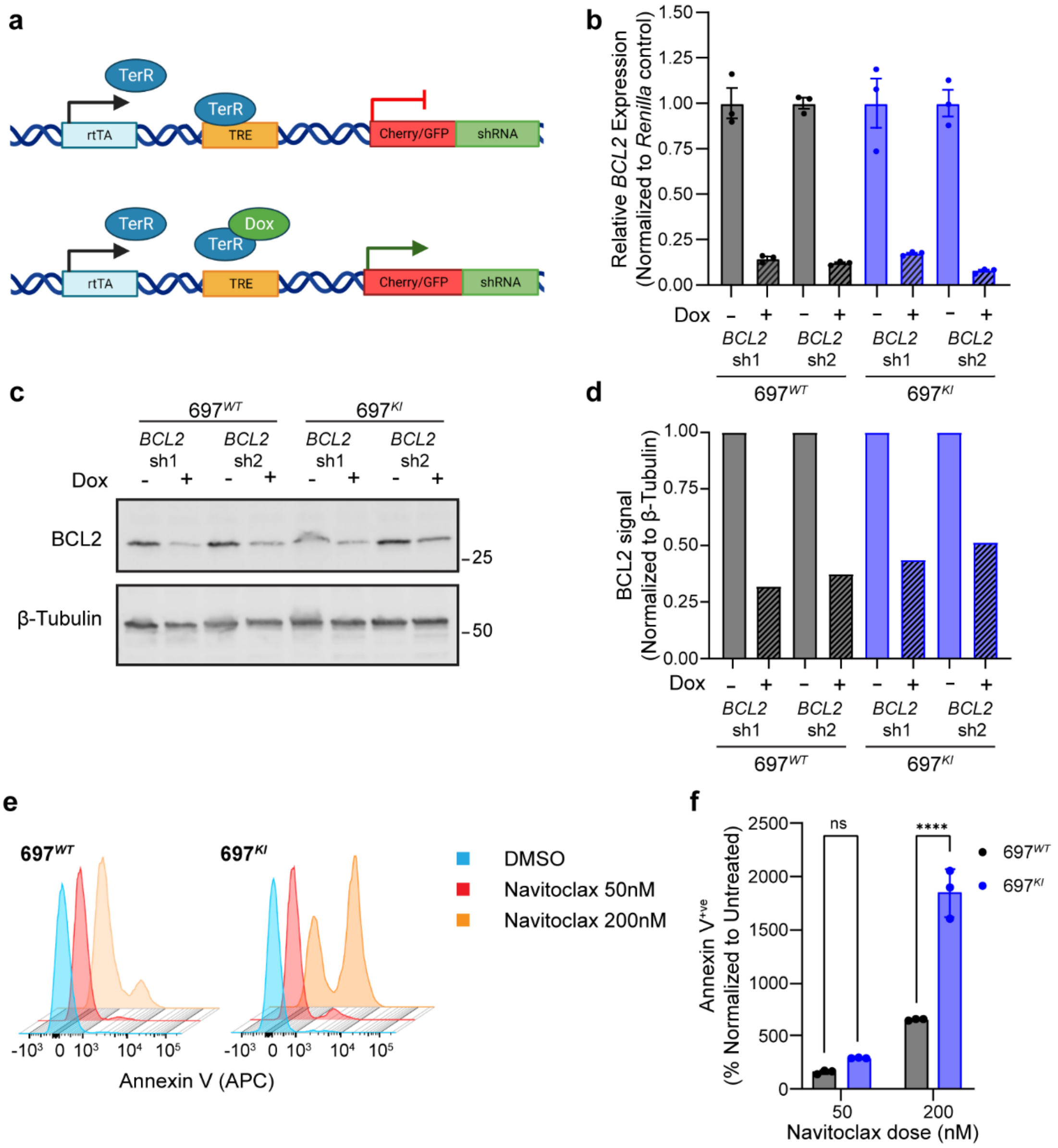

Supplementary Figure 2: Venetoclax exerts its effect on *CREBBP*-mutated B-ALL cell lines by on-target inhibition of BCL2.

a, Schematic of doxycycline-inducible shRNA KD system linked to fluorescent reporter proteins[24]. Created in BioRender. Huntly, B. (2025) https://BioRender.com/q87h680

b, Doxycycline-induced KD of two different *BCL2*-targeting shRNAs measured by RT-qPCR. n=3 technical replicates, internally normalised to *GAPDH* and presented as a ratio to *Renilla* control. Day 3 post induction. Mean±SEM.

c, Western blot of BCL2 KD by two different doxycycline-inducible shRNAs in 697^{WT} (left) and 697^{KI} (right) cells. Day 3 post induction. Beta-Tubulin is presented as a loading control.

d, Secondary antibody fluorescence intensity from Fig. S2C normalized to Beta-Tubulin loading control.

e, Representative flow cytometry histograms of Annexin-V (APC) externalization in response to escalating doses of Navitoclax in 697^{WT} (left) and 697^{KI} (right).

f, Summary of experiments in Fig. S2E measuring Annexin-V^{+ve} cells normalized to DMSO-treated vehicle control cells. n=3 independent replicates, mean±SD, 2-way ANOVA ****, *P*=0.0000025.

Source data are provided as a Source Data file.

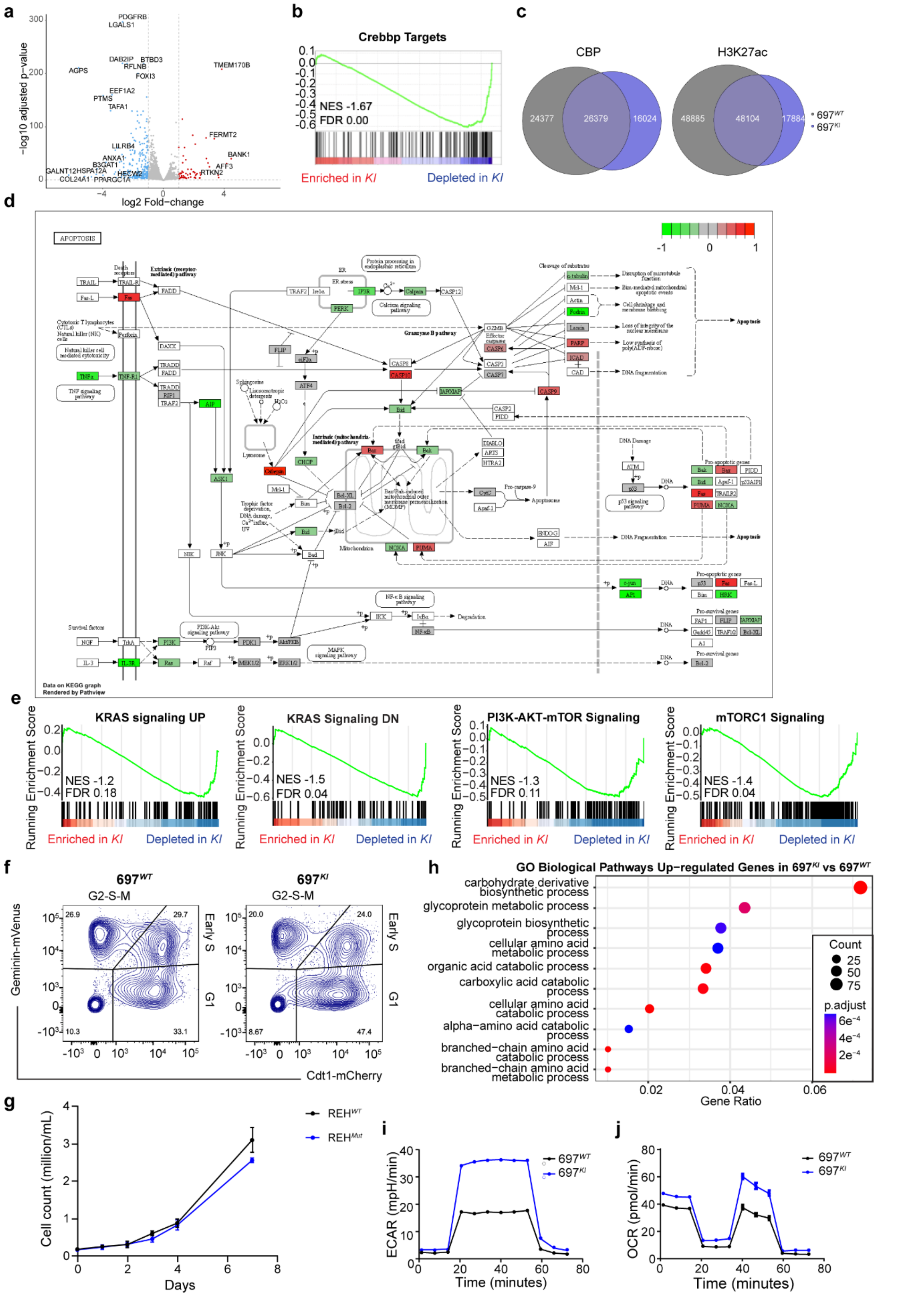

Supplementary Figure 3: *CREBBP*-mutated B-ALL cell lines show significant cell cycle and metabolic dysregulation.

a, Volcano plot of DEGs (P_{adj} and FDR <0.05, LFC >1) by RNAseq comparing 697^{KI} with 697^{WT} DMSO vehicle-treated cells.

b, GSEA of ranked RNAseq expression of 697^{KI} versus 697^{WT} DMSO vehicle-treated cells for known Crebbp target genes in *Crebbp*^{KO} mouse germinal centre lymphocytes[25].

c, Overlaps of CREBBP CUT&RUN binding sites (left) and H3K27ac ChIP marks (right) in 697^{WT} (black) and 697^{KI} (blue).

d, KEGG pathway showing differential expression of apoptotic regulators from RNAseq comparing 697^{KI} versus 697^{WT} DMSO vehicle-treated cells. Red genes upregulated, green downregulated.

e, GSEA of ranked RNAseq expression of 697^{KI} versus 697^{WT} DMSO vehicle-treated cells.

f, Representative plot of cell cycle stage by FUCCI reporter system in 697^{WT} (left) vs. 697^{KI} (right). Percentage viable single cells.

g, Proliferation of untreated REH^{WT} (black) and REH^{Mut} (blue) cells measured by direct counting. n=3 independent replicates, mean±SD.

h, Significant up-regulated pathways identified by Gene Ontology (GO) database analysis of RNAseq comparing 697^{KI} versus 697^{WT} DMSO vehicle-treated cells.

i, Glycolytic rate measured by extracellular acidification rate (ECAR) using Seahorse (Agilent) Glycostress test in 697^{WT} (black) and 697^{KI} (blue) cells. Representative ECAR plot over time. Mean±SEM.

j, Mitochondrial oxygen consumption rate (OCR) measured using Seahorse (Agilent) Mitostress test. Representative OCR plot over time. Mean±SEM.

Source data are provided as a Source Data file.

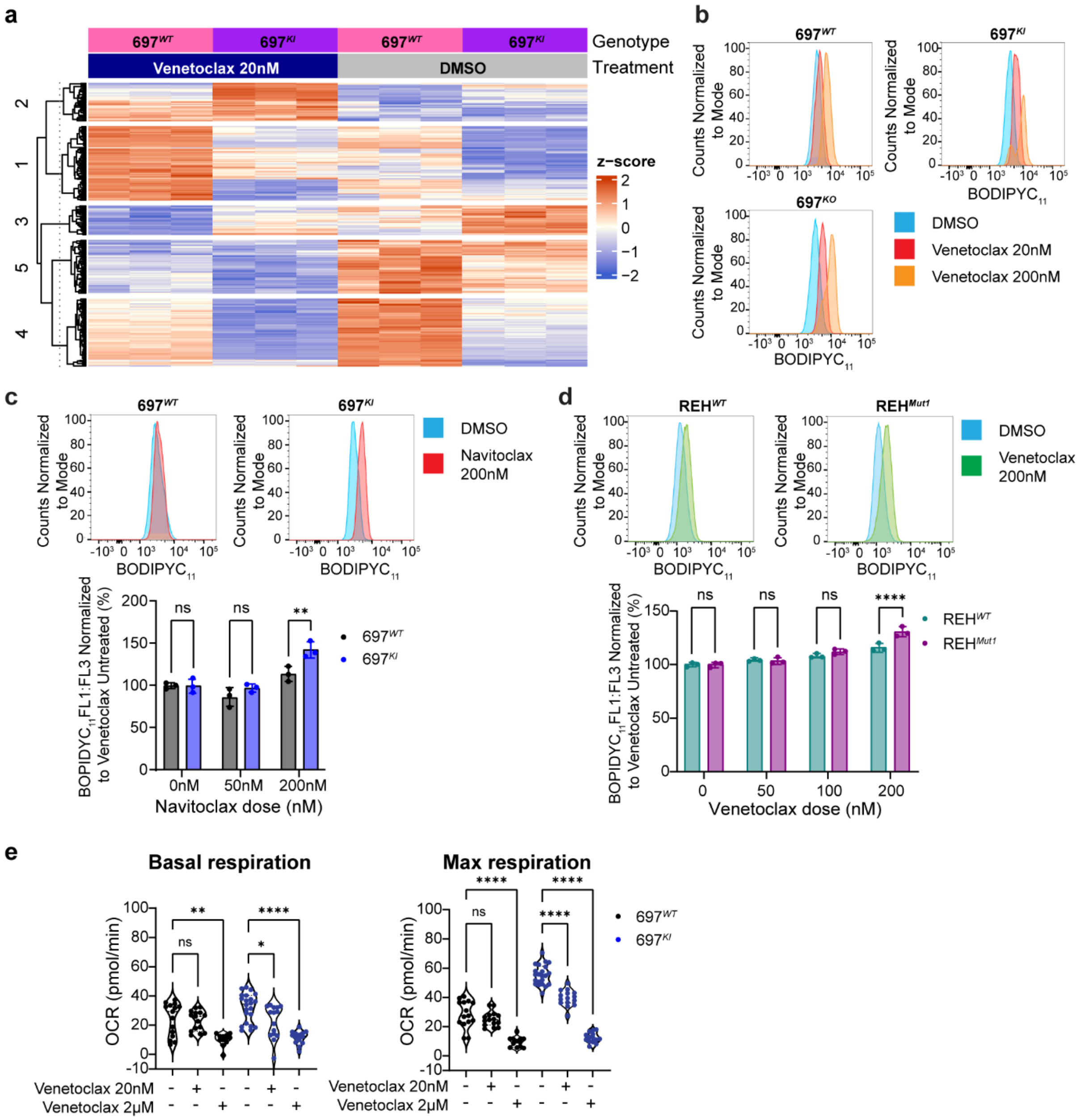

Supplementary Figure 4: Venetoclax induces ferroptotic cell death in *CREBBP*-mutated B-ALL cell lines.

a, Four-way interaction model identifies 1487 genes (FDR 0.05) differentially expressed specifically in Venetoclax-treated 697^{KI} cells.

b, Representative histogram of flow cytometric $BODIPYC_{11}$ staining (488nm 530/30) of 697^{WT} (left), 697^{KI} (right) and 697^{KO} (bottom) cells in response to increasing doses of Venetoclax (DMSO: blue; Venetoclax 20nM: red; Venetoclax 200nM: orange).

c, Top panel: representative histogram of flow cytometric $BODIPYC_{11}$ staining (488nm 530/30) of 697^{WT} (left) and 697^{KI} (right) cells in response to 200nM Navitoclax (red) or DMSO vehicle (blue). Bottom panel: summary $BODIPYC_{11}$ staining 697^{WT} (black) and 697^{KI} (blue) cells in response to 50nM Navitoclax, 200nM Navitoclax or DMSO vehicle normalized to DMSO vehicle. Independent triplicate, mean±SD, two-way ANOVA, **, P=0.0033

d, Top panel: representative histogram of flow cytometric BODIPYC_{11} staining (488nm 530/30) of REH^{WT} (left) and REH^{Mut1} (right) cells in response to 200nM of Venetoclax. Bottom panel: summary BODIPYC_{11} staining REH^{WT} (green) and REH^{Mut1} (purple) cells in response to Venetoclax normalized to DMSO vehicle. Independent triplicate, each dot represents a single sample, mean±SD, two-way ANOVA, ****, *P*=0.00005.

e, Summary of basal (left) and maximal (right) mitochondrial oxygen consumption rate (OCR) measured using Seahorse (Agilent) Mitostress test in 697^{WT} (black) and 697^{KI} (blue) cells exposed to increasing concentrations of Venetoclax. Each dot represents a single sample acquired from two separate experiments. One way ANOVA ****, Basal respiration: P=1.5x10^{-8}; Maximal respiration: 697^{WT} DMSO vs. 2µM P=3.6x10^{-10}, 697^{KI} DMSO vs. 20nM P=5.4x10^{-10}, 697^{KI} DMSO vs. 2µM P=3x10^{-10}; **, *P*=0.0012; *, *P*=0.0176 .

Source data are provided as a Source Data file.

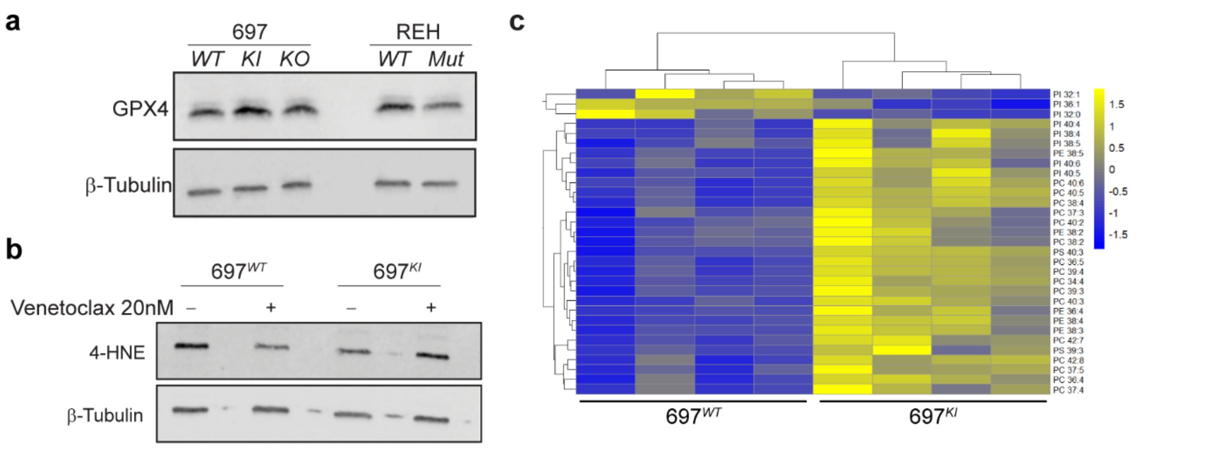

Supplementary Figure 5: CREBBP-mutation affects the redox balance and lipid content of B-ALL cell lines

a, Western blot comparing GPX4 expression in $697^{WT,\ KI,\ KO}$ (left) and $REH^{WT,\ Mut}$ (right). ß-tubulin loading control.

b, Western blot of 4-hydroxyneonal (4-HNE) adducts in 697^{WT} and 697^{KI} in presence of DMSO control or low dose Venetoclax (20nM). ß-tubulin loading control.

c, Hierarchically clustered heatmap of selected phosphatidylinositol (PI), phosphatidylethanolamine (PE) and phosphatidylcholine (PC) lipids that differ significantly between 697^{WT} (left) and 697^{KI} (right). n=4 independent replicates. Relative normalized expression.

Source data are provided as a Source Data file.

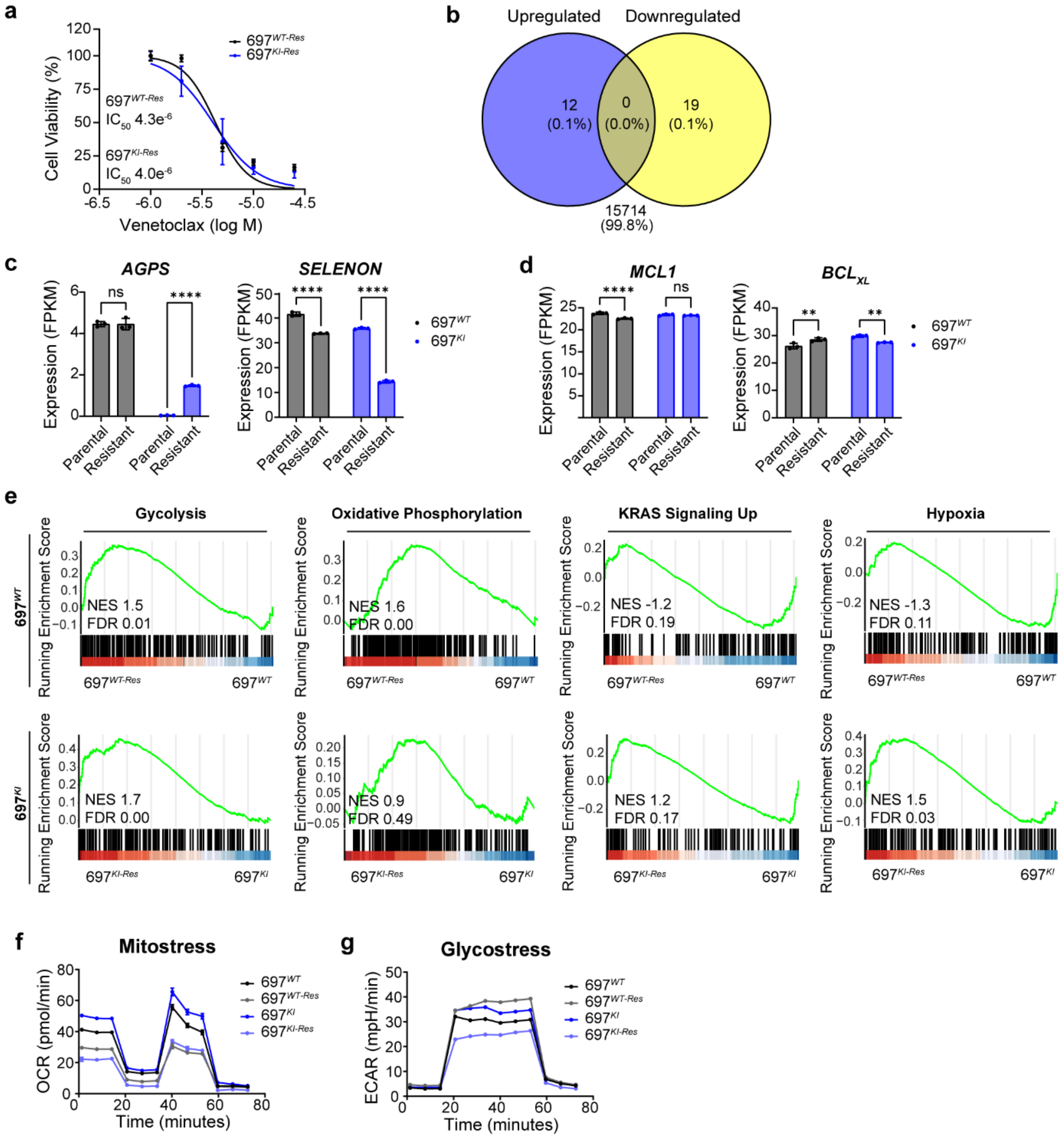

Supplementary Figure 6: Acquisition of Venetoclax resistance results in transcriptional convergence

a, Dose response curve of $697^{WT\text{-Res}}$ (black) and $697^{KI\text{-Res}}$ (blue) to Venetoclax in 72h MTS viability assays. n=3 technical replicates, mean±SD.

b, Number of up- and down-regulated DEGs (LFC>1) by RNAseq comparing $697^{WT\text{-Res}}$ and $697^{KI\text{-Res}}$ cells.

c, Comparison of *AGPS* (left) and *SELENON* (right) expression levels by RNAseq in 697^{WT} (black) and 697^{KI} (blue) cells comparing parental and Venetoclax-resistant lines. Each dot represents one sample. Bars show mean FPKM value±SD, n=3 independent replicates. Two-way ANOVA, ****, *AGPS* $P=6\times10^{-6}$, *SELENON* 697^{WT} $P=1.3\times10^{-7}$, 697^{KI} $P=3.9\times10^{-11}$.

d, Comparison of *MCL1* (left) and BCL_{XL} (*BCL2L1* gene) (right) expression levels by RNAseq in 697^{WT} (black) and 697^{KI} (blue) cells comparing parental and Venetoclax-resistant lines. Each dot represents

one sample. Bars show mean FPKM value±SD, n=3 independent replicates. Two-way ANOVA, ****, P=8.6x10^{-5}; **, P=0.0045.

e, GSEA of ranked RNAseq expression comparing $697^{WT\text{-Res}}$ (left) and $697^{KI\text{-Res}}$ (right) with their matched parental lines.

f, Representative mitochondrial oxygen consumption rate (OCR) measured using Seahorse (Agilent) Mitostress test in 697^{WT} (black) and 697^{KI} (blue) cells comparing parental and resistant lines. Mean±SEM.

g, Representative plot of glycolytic rate measured by extracellular acidification rate (ECAR) using Seahorse (Agilent) Glycostress test in 697^{WT} (black) and 697^{KI} (blue) cells comparing parental and resistant lines. Mean±SEM.

Source data are provided as a Source Data file.

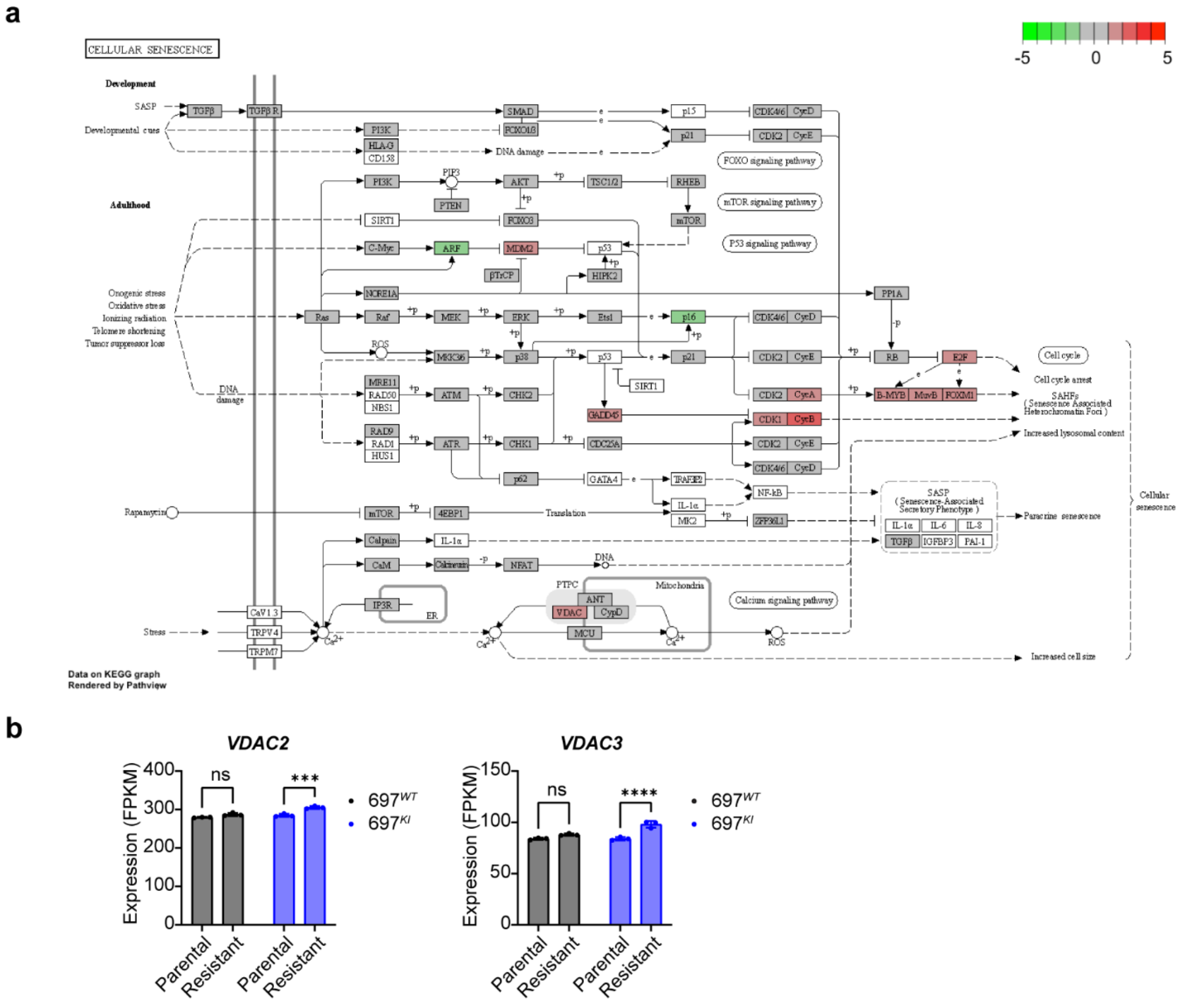

Supplementary Figure 7: Acquisition of Venetoclax resistance sensitizes cells to Erastin.

a, KEGG pathway analysis showing differential expression of cellular senescence regulators from RNAseq comparing $697^{KI\text{-Res}}$ vs. 697^{KI} parental cells (FDR $q=3\text{x}10^{-10}$).

b, Comparison of *VDAC2* (left) and *VDAC3* (right) expression levels by RNAseq in 697^{WT} (black) and 697^{KI} (blue) cells comparing parental and Venetoclax resistant lines. Each dot represents one sample. Bars show mean FPKM value±SD, n=3 independent replicates. Two-way ANOVA, ****, P=4x10^{-5}; ***, P=0.0002.

Source data are provided as a Source Data file.

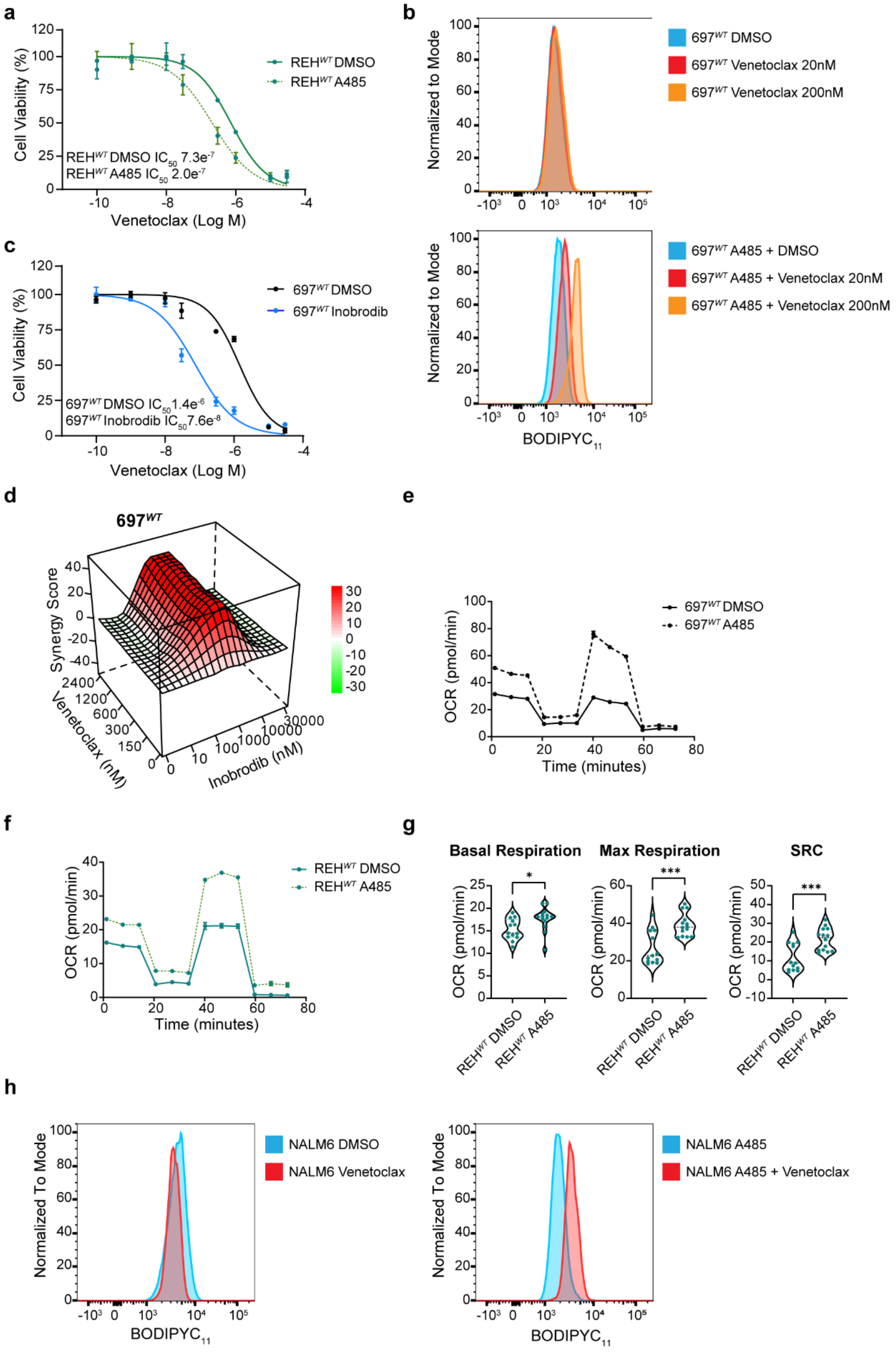

a
Cell Viability (%)
Venetoclax (Log M)
REH^WT DMSO
REH^WT A485
REH^WT DMSO IC50 7.3e-7
REH^WT A485 IC50 2.0e-7
b
Normalized to Mode
697^WT DMSO
697^WT Venetoclax 20nM
697^WT Venetoclax 200nM
697^WT A485 + DMSO
697^WT A485 + Venetoclax 20nM
697^WT A485 + Venetoclax 200nM
BODIPYC11
c
697^WT DMSO
697^WT Inobrodib
697^WT DMSO IC50 1.4e-6
697^WT Inobrodib IC50 7.6e-8
d
697^WT
Synergy Score
Venetoclax (nM)
Inobrodib (nM)
e
OCR (pmol/min)
Time (minutes)
697^WT DMSO
697^WT A485
f
REH^WT DMSO
REH^WT A485
g
Basal Respiration
Max Respiration
SRC
h
Normalized To Mode
NALM6 DMSO
NALM6 Venetoclax
NALM6 A485
NALM6 A485 + Venetoclax

Supplementary Figure 8: Pharmacological inhibition of CREBBP function can sensitize B-ALL cell lines to Venetoclax *in-vitro*.

a, Dose response curve of REH^{WT} (solid line) and REH^{WT} pre-treated with 3 days of A485 (hashed line) to Venetoclax in 72 hour MTS viability assays. n=3 technical replicates, mean±SD.

b, Representative histogram of flow cytometric $BODIPYC_{11}$ staining of 697^{WT} (top) and 697^{WT} pre-treated with A485 (bottom) in response to increasing doses of Venetoclax.

c, Dose response curve of 697^{WT} (black) and 697^{WT} pre-treated with 3 days of Inobrodib (pale blue) to Venetoclax in 72h MTS viability assays. n=3 technical replicates, mean±SD.

d, Three-dimensional diffusion plot of ZIP synergy score to combined doses of synchronous Inobrodib and Venetoclax (peak ZIP 37.58). Viability measured by 72h MTS assay.

e, Representative mitochondrial oxygen consumption rate (OCR) measured using Seahorse (Agilent) Mitostress test in 697^{WT} (solid line) compared to 697^{WT} treated with A485 (hashed line). Mean±SEM.

f, Representative mitochondrial oxygen consumption rate (OCR) measured using Seahorse (Agilent) Mitostress test in REH^{WT} (solid line) compared to REH^{WT} treated with A485 (hashed line). Mean±SEM.

g, Summary of basal (left) and maximal (middle) mitochondrial oxygen consumption rate (OCR) and spare respiratory capacity (right) measured using Seahorse (Agilent) Mitostress test in REH^{WT} cells treated with A485 or DMSO vehicle. Each dot represents a single replicate acquired from two separate experiments. Unpaired *t*-test, ***, P >0.0001; *, P=0.0198.

h, Representative histogram of flow cytometric $BODIPYC_{11}$ staining of $NALM6^{WT}$ (left) and $NALM6^{WT}$ pre-treated with A485 (right) in response to Venetoclax.

Source data are provided as a Source Data file.

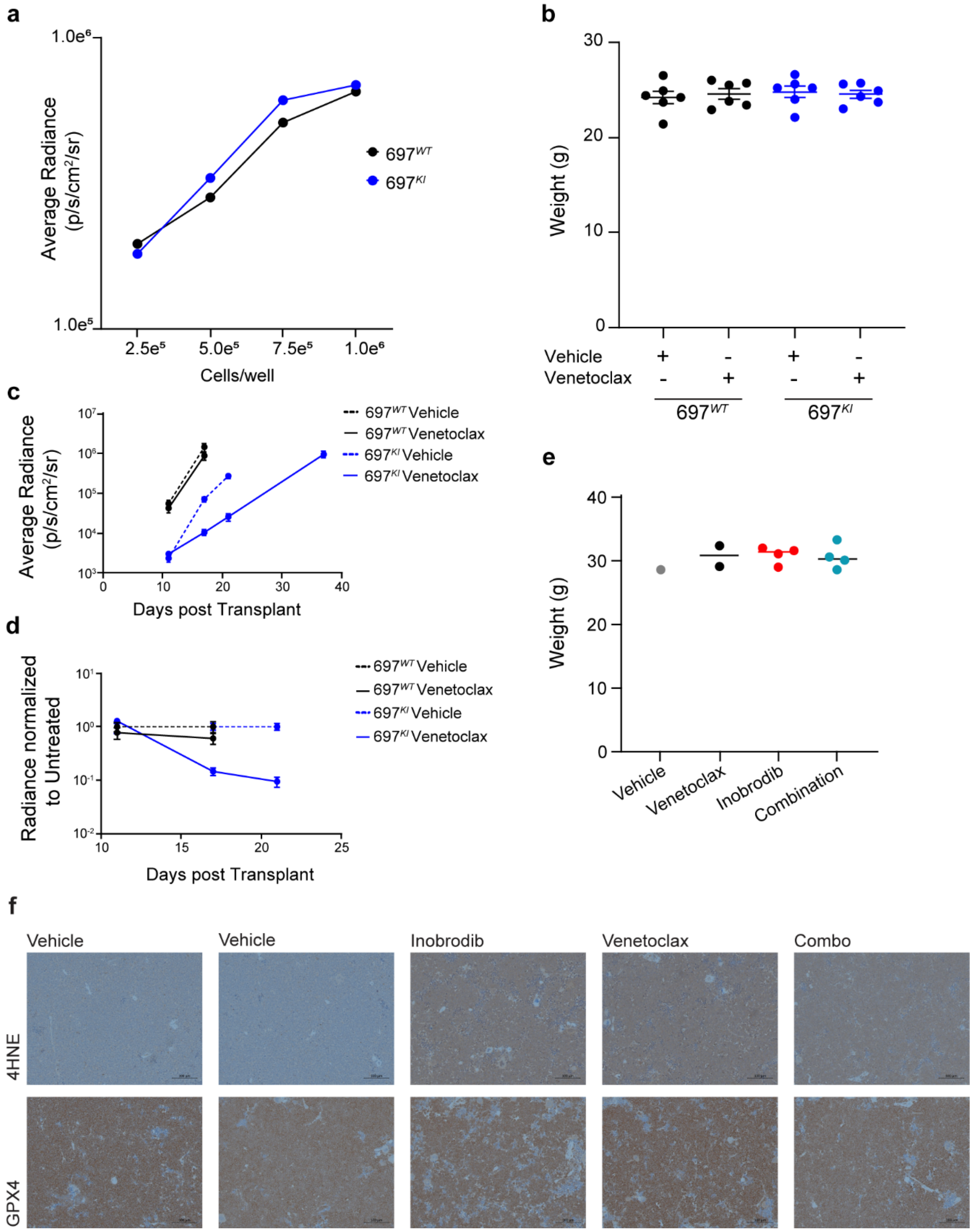

Supplementary Figure 9: Genetic or pharmacological inhibition of CREBBP sensitizes B-ALL to Venetoclax *in-vivo*.

a, *In-vitro* BLI measurements of a serial dilution of luciferase-expressing 697^{WT} vs. 697^{KI} B-ALL cells (log p/s/cm^2/sr).

b, Baseline weights of NSG mice treated in Fig. 7A-F. Mean±SEM.

c, Average BLI radiance of mice engrafted with 697^{WT} or 697^{KI} cells treated with Venetoclax or vehicle control at days 11, 17 and 21 and 37 (697^{KI} only) of treatment (log p/s/cm^2/sr). Mean±SEM. n=6 mice per group (n=5 in 697^{KI} Vehicle due to imaging failure, n=5 697^{KI} Venetoclax D37 due to one prior death).

d, Average BLI radiance of mice engrafted with 697^{WT} or 697^{KI} cells treated with Venetoclax or vehicle control at days 11, 17 and 21 (697^{KI} only) of treatment (log p/s/cm^2/sr) normalized to untreated 697^{KI} recipients. Mean±SEM. n=6 mice per group (n=5 in 697^{KI} Vehicle due to imaging failure).

e, Baseline weights of NSG mice treated in Fig. 7G. Bar represents median average.

f, Immunohistochemical staining for 4-HNE adducts (top) and GPX4 protein expression (bottom) in B-ALL PDX-infiltrated spleens from NSG mice, comparing two Vehicle treated control mice (left & centre left) with mice acutely treated with 2 doses of Inobrodib (centre), Venetoclax (centre right), or combination (right) treatment.

Source data are provided as a Source Data file.

Gating strategy for JC1 (Mitochondrial depolarisation)

Figure 1F

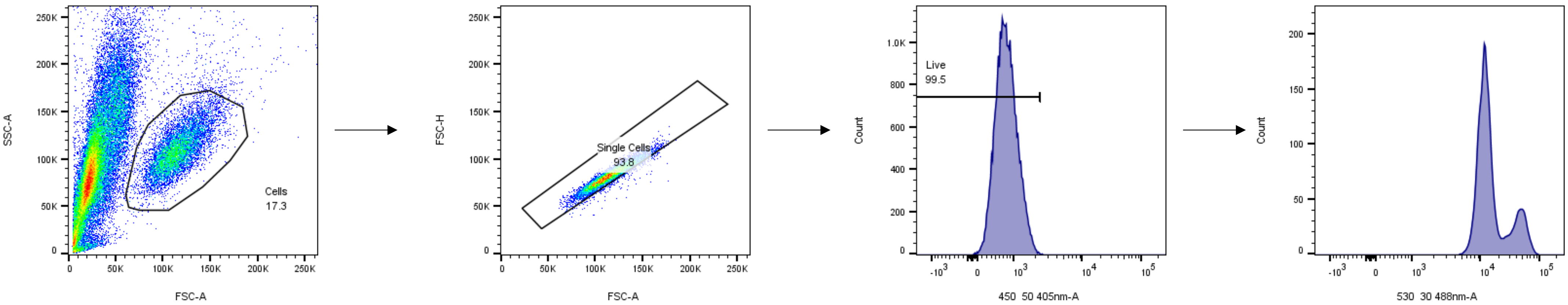

Gating strategy for Annexin V APC

Figure 1G, H; 2C, D; 6F

Supplementary figures 2E, F

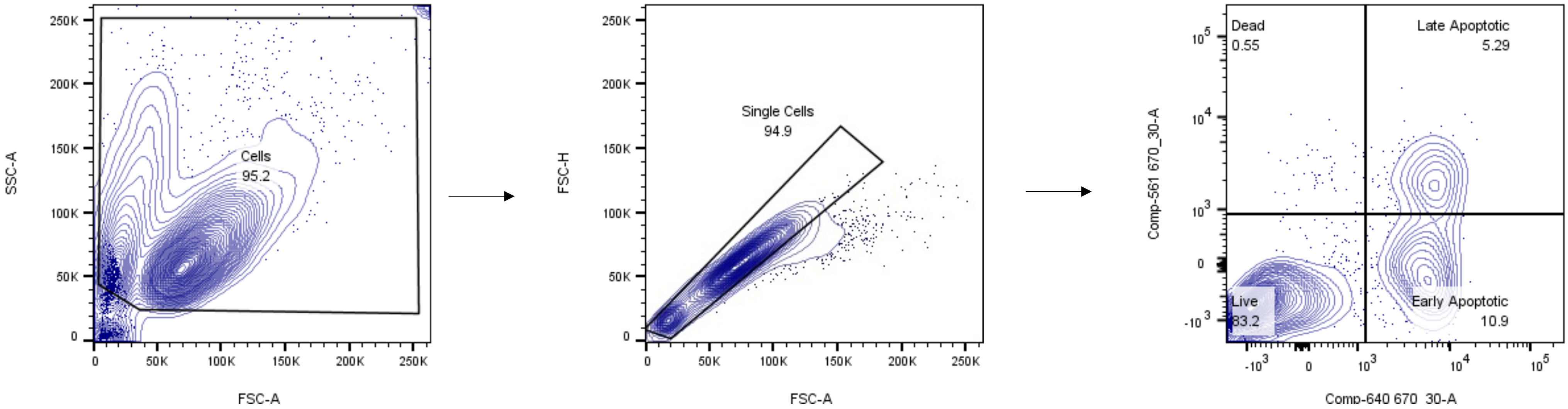

Gating streategy for shRNA fluorescent reporting proteins.

Figure 2B

Supplementary figure 2B

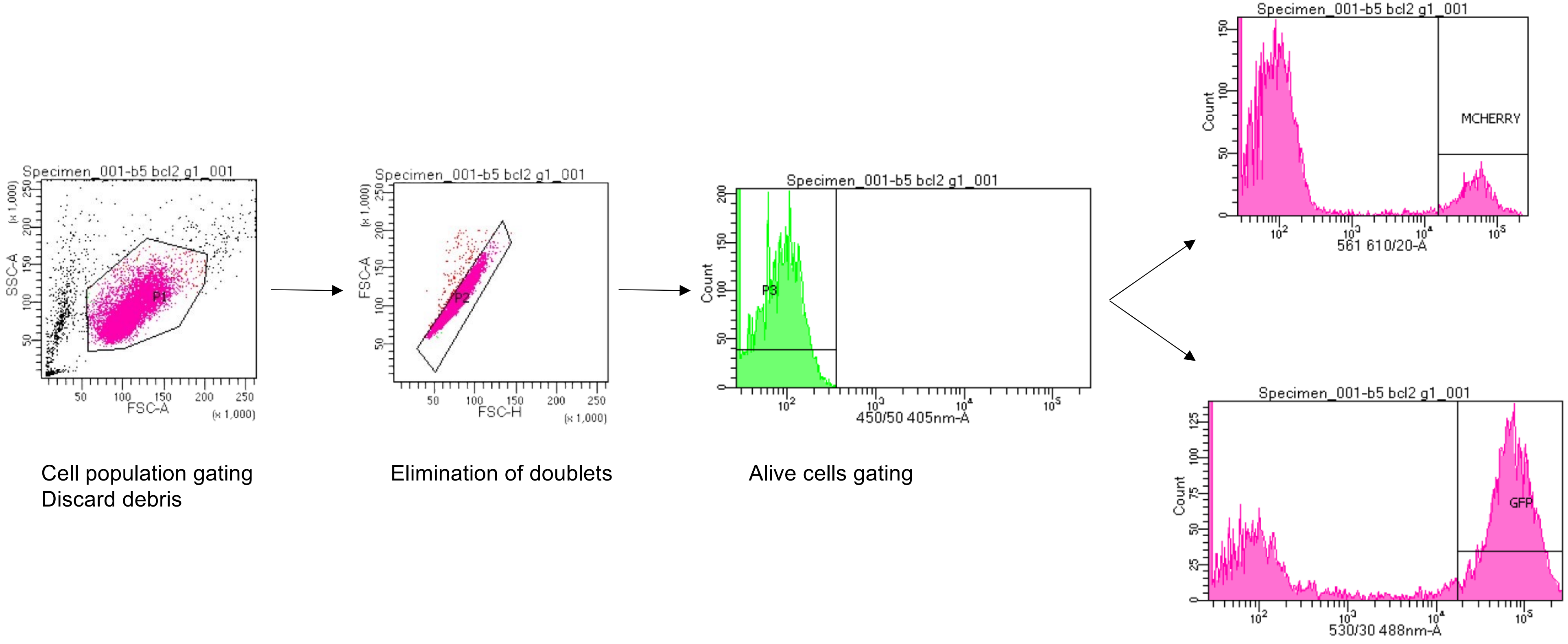

Cell population gating
Discard debris

Elimination of doublets

Alive cells gating

Gating of fluorescent reporters

Gating strategy for Fucci reporter system

Figure 3G

Supplementary figures 3F

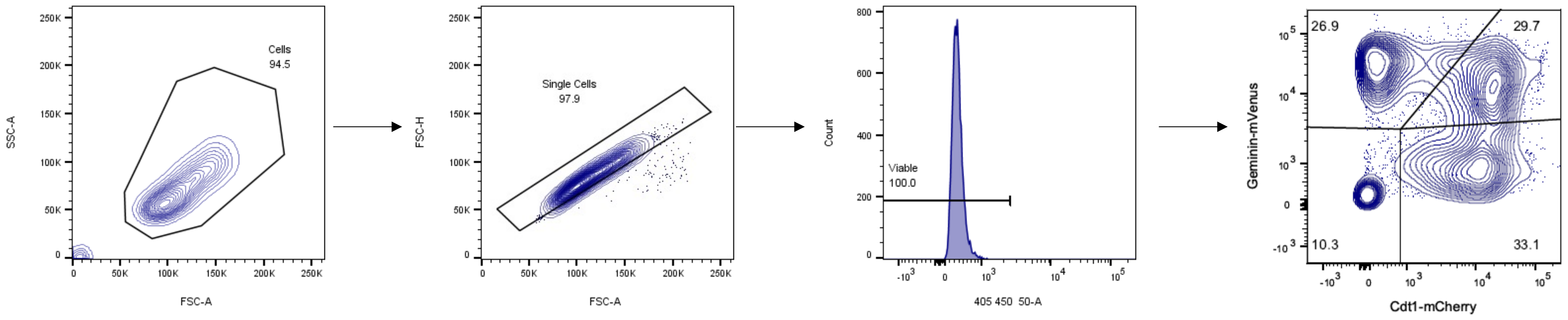

Gating strategy for BODIPYC11

Figure 4E, G; 5B; 6C, H;

Supplementary figures 4B, C, D; 6B, H

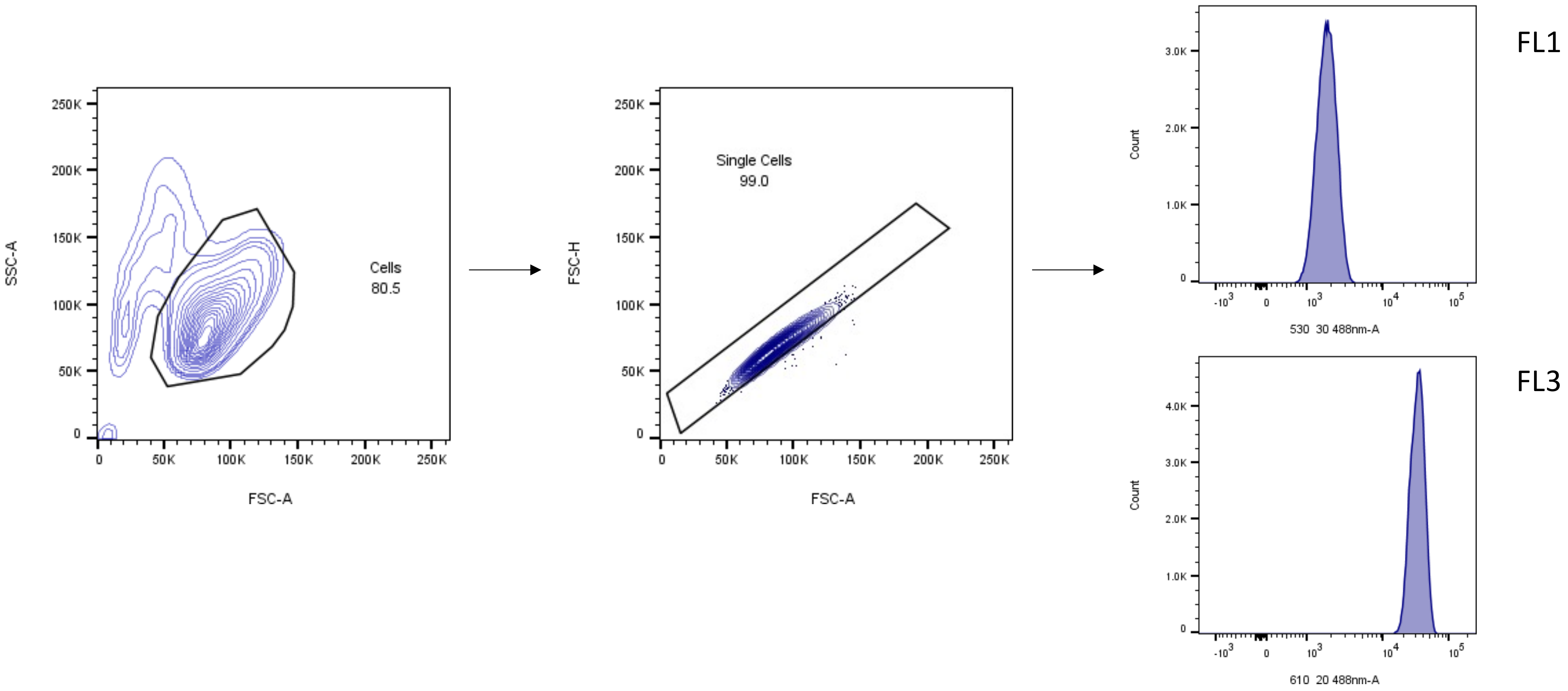

Supplement: Supplementary file 1 — Supplementary Information [file 41467_2025_59531_MOESM1_ESM.pdf]
