# Supplementary material for: CREBBP inactivation sensitizes B cell acute lymphoblastic leukemia to ferroptotic cell death upon BCL2 inhibition
Source: Nat Commun. 2025 May 20;16:4274. doi: 10.1038/s41467-025-59531-6 (PMC12092839; doi:10.1038/s41467-025-59531-6)
Supplement: Supplementary file 2 — Description of Additional Supplementary Information [file 41467_2025_59531_MOESM2_ESM.docx]

**Description of Additional Supplementary Information:**

**Supplementary Data 1:** Cell viability data from small molecule screen in 697*^WT^*, 697*^KI^* and 697*^KO^* cell lines (related to figure 1). One-way Brown-Forsythe and Welch ANOVA test with Dunnett T3 comparison comparing to 697*^WT^* used for statistical analysis, unless specified with a * where unpaired T-test used.

**Supplementary Data 2:** Differentially regulated proteins. Two-sided limma statistical test. To control for the false discovery rate (FDR), p-values were adjusted using the Benjamini-Hochberg method for multiple testing correction.

**Supplementary Data 3:** Lipidomics raw data, standards and parameters.

**Supplementary Data 4:** Two-sided Pearson correlation with p-value adjusted for multiple testing using Bonferroni correction of gene expression with *CREBBP* expression in the TARGET phase 2 RNAseq cohort. Gene sets for ferroptosis are shown.
