# Supplementary material for: CREBBP inactivation sensitizes B cell acute lymphoblastic leukemia to ferroptotic cell death upon BCL2 inhibition
Source: Nat Commun. 2025 May 20;16:4274. doi: 10.1038/s41467-025-59531-6 (PMC12092839; doi:10.1038/s41467-025-59531-6)
Supplement: Supplementary file 7 — Reporting Summary [file 41467_2025_59531_MOESM7_ESM.pdf]

nature portfolio

Corresponding author(s): Brian J.P Huntly
Simon E. Richardson

Last updated by author(s): 26/3/25

Reporting Summary

Nature Portfolio wishes to improve the reproducibility of the work that we publish. This form provides structure for consistency and transparency in reporting. For further information on Nature Portfolio policies, see our Editorial Policies and the Editorial Policy Checklist.

Statistics

For all statistical analyses, confirm that the following items are present in the figure legend, table legend, main text, or Methods section.

n/a	Confirmed	
☐	☒	The exact sample size (*n*) for each experimental group/condition, given as a discrete number and unit of measurement
☐	☒	A statement on whether measurements were taken from distinct samples or whether the same sample was measured repeatedly
☐	☒	The statistical test(s) used AND whether they are one- or two-sided<br>*Only common tests should be described solely by name; describe more complex techniques in the Methods section.*
☐	☒	A description of all covariates tested
☐	☒	A description of any assumptions or corrections, such as tests of normality and adjustment for multiple comparisons
☐	☒	A full description of the statistical parameters including central tendency (e.g. means) or other basic estimates (e.g. regression coefficient) AND variation (e.g. standard deviation) or associated estimates of uncertainty (e.g. confidence intervals)
☐	☒	For null hypothesis testing, the test statistic (e.g. *F*, *t*, *r*) with confidence intervals, effect sizes, degrees of freedom and *P* value noted<br>*Give P values as exact values whenever suitable.*
☒	☐	For Bayesian analysis, information on the choice of priors and Markov chain Monte Carlo settings
☒	☐	For hierarchical and complex designs, identification of the appropriate level for tests and full reporting of outcomes
☐	☒	Estimates of effect sizes (e.g. Cohen's *d*, Pearson's *r*), indicating how they were calculated

Our web collection on statistics for biologists contains articles on many of the points above.

Software and code

Policy information about availability of computer code

Data collection	Wave (2.6.3), BD FACSDiva (8.0.1), MARS (3.42), Image Studio (4.0), MxPro Mx3000P (4.10), MS-DIAL software (4.9).
Data analysis	Microsoft Excel, SynergyFinder, FlowJo (10), GraphPad Prism (9), STAR (2.7.10a), Living Image Software (4.7.2), LipidOne (2.0), Rstudio packages: ClusterProfiler (4.4.4), AnnotationHub (3.4.0), ComplexHeatmap (2.12.1), enrichplot (1.18.4), ensembldb (2.20.2), ggplot2 (3.4.2), ggvenn (0.1.10), msigdbr (7.5.1), org.Hs.eg.db (3.15.0), pathview (1.36.1), sva (3.44.0), MACS2 (2.2.7.1), bedops (2.4.41), ChIPseeker.

For manuscripts utilizing custom algorithms or software that are central to the research but not yet described in published literature, software must be made available to editors and reviewers. We strongly encourage code deposition in a community repository (e.g. GitHub). See the Nature Portfolio guidelines for submitting code & software for further information.

Data

Policy information about availability of data

All manuscripts must include a data availability statement. This statement should provide the following information, where applicable:

- Accession codes, unique identifiers, or web links for publicly available datasets
- A description of any restrictions on data availability
- For clinical datasets or third party data, please ensure that the statement adheres to our policy

Raw sequencing data for RNAseq, ChIPseq and CUR&RUN are deposited in GEO Archive under accession numbers GSE248265, GSE289011 and GSE289012 respectively [https://www.ncbi.nlm.nih.gov/geo/]. The total proteomics data generated in this study have been deposited in the ProteomeXchange database under accession code PXD061362 [https://www.ebi.ac.uk/pride/archive?sortDirection=DESC&page=0&pageSize=20]. The total lipidomics data generated in this study have

been deposited in the MassIVE database under accession code MSV000097461 [https://massive.ucsd.edu/ProteoSAFe/dataset.jsp?task=6746d9c3cb2d4c4e957cde41bd03aa08]. Further information and requests for resources and reagents should be directed to and will be fulfilled by the corresponding author. Source data are provided with this paper.

Research involving human participants, their data, or biological material

Policy information about studies with human participants or human data. See also policy information about sex, gender (identity/presentation), and sexual orientation and race, ethnicity and racism.

Reporting on sex and gender	N/A
Reporting on race, ethnicity, or other socially relevant groupings	N/A
Population characteristics	N/A
Recruitment	N/A
Ethics oversight	PDX material was generated from a genotyped, male, pediatric patient with primary cells sourced from the VIVO biobank (REC reference 23/EM/0130). Parental consent was obtained and no compensation provided.

Note that full information on the approval of the study protocol must also be provided in the manuscript.

Field-specific reporting

Please select the one below that is the best fit for your research. If you are not sure, read the appropriate sections before making your selection.

- [x] Life sciences
- [ ] Behavioural & social sciences
- [ ] Ecological, evolutionary & environmental sciences

For a reference copy of the document with all sections, see nature.com/documents/nr-reporting-summary-flat.pdf

Life sciences study design

All studies must disclose on these points even when the disclosure is negative.

Sample size	Sample sizes were based on pilot studies showing median survival times for 697 cells. For the Venetoclax dosing studies in Figure 9a-f, a sample size of 6 was chosen to detect a 5 ± 3 day OS benefit in the treatment arm, based on an alpha of 0.05 and a power of 80% and an anticipated engraftment time of 24 days in the control group. Recipients were 13-15 week-old female NSG (NOD.Cg-Prkdcscid Il2rgtm1Wjl/SzJ). Animals were randomized by weight.<br>For the combination studies in Figure 9g, minimal control numbers were used for venetoclax or vehicle dosing in line with three R principles. The number of mice used for treatment of either Inobrodib or Inobrodib with Venetoclax was calculated as four per arm, based on an anticipated survival benefit of 6 ± 3 days in the dual treatment arm, based on an alpha of 0.05 and a power of 80% and an anticipated engraftment time of 24 days in the control group. Power calculations were calculated using ClinCalc.com. Recipients were 11-13 week-old old male NSG (NOD.Cg-Prkdcscid Il2rgtm1Wjl/SzJ) mice. Animals were randomized by weight.<br>For the combination dosing studies in PDX in Figure 9h a minimal sample size of 5 was chosen to detect a 35 ± 20 day OS benefit in the treatment arms, based on an alpha of 0.05 and a power of 80% and a predicted latency of 120 days. Recipients were 17-23 week-old old male and female NSG (NOD.Cg-Prkdcscid Il2rgtm1Wjl/SzJ) mice. Animals were randomized by sex and weight. Recipients were 17-23 week-old old male and female NSG mice. Animals were randomized by sex and weight (Vehicle 2M/3F; Inobrodib 2M/3F; Venetoclax 2M/5F; Combination 3M/4F).
Data exclusions	One mouse engrafted with 697KI treated with vehicle failed IVIS and was excluded from fig 9e and figs S9c/d. No other data from the in vivo experiments was excluded.<br>1/5 replicates from the lipidomics was an outlier on global analysis and clearly different to all other samples in the experiment. Whilst inclusion of this data did not change the overall conclusions or significance, it was felt to be unrepresentative of therefore excluded.<br>Seahorse experiments were performed with multiple biological replicates on at least two separate occasions to account for technical failures or gross outliers, which were excluded based on the independent judgment of two researchers.
Replication	All attempts at replication were successful.
Randomization	Animals were allocated to receive 697 WT / KI cells based on weight. Treatment arms were weight and sex matched.
Blinding	The investigator was not blinded to the group allocation, however, the animal technicians who provided the majority of animal care and who decided upon which animals to sacrifice (according to very strict guidelines) were blinded to the allocation. Mice showing any clinical signs or weight loss (more than 20% of its reference weight post irradiation or 15% after administration of therapeutics), or sustained signs of compromised wellbeing such as reduced activity, piloerection and hunching that persisted for more than 6 hours (or 2 health checks separated by at least three hours) were humanely sacrificed.

Reporting for specific materials, systems and methods

We require information from authors about some types of materials, experimental systems and methods used in many studies. Here, indicate whether each material, system or method listed is relevant to your study. If you are not sure if a list item applies to your research, read the appropriate section before selecting a response.

Materials & experimental systems

n/a	Involved in the study
☐	☒ Antibodies
☐	☒ Eukaryotic cell lines
☒	☐ Palaeontology and archaeology
☐	☒ Animals and other organisms
☐	☒ Clinical data
☒	☐ Dual use research of concern
☒	☐ Plants

Methods

n/a	Involved in the study
☐	☒ ChIP-seq
☐	☒ Flow cytometry
☒	☐ MRI-based neuroimaging

Antibodies

Antibodies used

Lot numbers listed where available.
DAPI (BD, 564907) 1/10000
7AAD (BD, 559925), 1/100 or 1/500
Caspase 3/PARP (Abcam, ab136812, Lot: 2101026931), 1/250
BCL2 (E17) (Abcam, ab32124, Lot: GR3232704-4) 1/1000
β-tubulin (Sigma-Aldrich, T8328), 1/2000
CREBBP (A22)(Santa Cruz biotechnology, SC369, Lot: G1613) 1µg for CUTandRUN, 1/500 for WB
Vinculin (H-10) (Santa Cruz biotechnology, sc25336, Lot: I2718), 1/250
4HNE (Abcam, ab46545, polyclonal, Lot: 1073022-2), 1/200 for IHC,1/1000 for WB
GPX4 (Abcam, ab41787, polyclonal, Lot: 1047829-4), 1/200 for IHC, 1/1000 for WB
IgG (Proteintech, 30000-0-AP), 2.5µg
H3 (1B1B2) (Cell Signalling Technology, 14269S), 2.5µg
H3K27ac (Active motif, 39133), 2.5µg
IgG (Epicypher, cat 13-0042), 1µg

Validation

All antibodies used are commercially available and have been validated by the manufacturer.
Specifically, antibodies used for ChIP and CUTandRUN were validated for these applications by Active motif, EpiCypher and cell signalling technologies. Abcam antibodies to BCL2 and GPX4 have been commercially validated using KO.

Eukaryotic cell lines

Policy information about cell lines and Sex and Gender in Research

Cell line source(s)	B-ALL cell lines 697 (ACC 42), REH (ACC 22) and NALM6 (ACC 128) cell lines were purchased from DSMZ. RS411, KOPN8 and SUPB15 were provided by Prof. Owen Williams (UCL, UK). 697, NALM6 and SUPB15 cell lines derive from male individuals while REH, RS411 and KOPN8 derive from female individuals.
Authentication	STR genotyping was performed on all cell lines by Promega Powerplex 16-HS kit (performed by Genetica LabCorp).
Mycoplasma contamination	Cell lines were regularly tested negative for mycoplasma negativity by PCR (CSCI Core Facility).
Commonly misidentified lines (See ICLAC register)	No commonly misidentified lines were used.

Animals and other research organisms

Policy information about studies involving animals; ARRIVE guidelines recommended for reporting animal research, and Sex and Gender in Research

Laboratory animals	All animals were NSG (NOD.Cg-Prkdcscid Il2rgtm1Wjl/SzJ) mice originally sourced and colony refreshed from Charles River.
Wild animals	N/A
Reporting on sex	Female mice were used for cell line in vivo dosing experiments in fig 9a-f. Male mice were used for in vivo dosing in fig 9g. A mix of male and female mice were used for PDX experiments in fig 9h.
Field-collected samples	N/A
Ethics oversight	All experiments were conducted under a UK Home Office project (under the Animals (Scientific Procedures) Act 1986, Amendment Regulations (2012)) and following ethical review by the University of Cambridge Animal Welfare and Ethical Review Body.

Note that full information on the approval of the study protocol must also be provided in the manuscript.

Clinical data

Policy information about clinical studies
All manuscripts should comply with the ICMJEguidelines for publication of clinical research and a completedCONSORT checklist must be included with all submissions.

Clinical trial registration	N/A
Study protocol	N/A
Data collection	N/A
Outcomes	N/A

Plants

Seed stocks	N/A
Novel plant genotypes	N/A
Authentication	N/A

ChIP-seq

Data deposition

- [x] Confirm that both raw and final processed data have been deposited in a public database such as GEO.
- [x] Confirm that you have deposited or provided access to graph files (e.g. BED files) for the called peaks.

Data access links<br>*May remain private before publication.*	GSE289012<br>GSE289011
Files in database submission	CREBBP2_4B7_CutNRun_R1_001.fastq.gz<br>CREBBP2_4B7_CutNRun_R2_001.fastq.gz<br>CREBBP2_4B7_CutNRun.cpm.norm.bw<br>CREBBP2_4B7_CutNRun.input_peaks.narrow_0.05_FDR.bed<br>CREBBP2_B5_CutNRun_R1_001.fastq.gz<br>CREBBP2_B5_CutNRun_R2_001.fastq.gz<br>CREBBP2_B5_CutNRun.cpm.norm.bw<br>CREBBP2_B5_CutNRun.input_peaks.narrow_0.05_FDR.bed<br>H3K27ac_4B7_ChIP_R1_001.fastq.gz<br>H3K27ac_4B7_ChIP_R2_001.fastq.gz<br>H3K27ac_4B7_ChIP.cpm.norm.bw<br>H3K27ac_4B7_ChIP.input_peaks.narrow_0.05_FDR.bed<br>H3K27ac_B5_ChIP_R1_001.fastq.gz<br>H3K27ac_B5_ChIP_R2_001.fastq.gz<br>H3K27ac_B5_ChIP.cpm.norm.bw<br>H3K27ac_B5_ChIP.input_peaks.narrow_0.05_FDR.bed<br>Input_4B7_CutNRun.cpm.norm.bw<br>Input_4B7_CutNRun_R1_001.fastq.gz<br>Input_4B7_CutNRun_R2_001.fastq.gz<br>Input_B5_CutNRun.cpm.norm.bw<br>Input_B5_CutNRun_R1_001.fastq.gz<br>Input_B5_CutNRun_R2_001.fastq.gz<br>Input_4B7_ChIP_R1_001.fastq.gz<br>Input_4B7_ChIP_R2_001.fastq.gz<br>Input_4B7_ChIP.cpm.norm.bw<br>Input_B5_ChIP_R1_001.fastq.gz<br>Input_B5_ChIP_R2_001.fastq.gz<br>Input_B5_ChIP.cpm.norm.bw

Genome browser session
(e.g. UCSC)

https://tinyurl.com/28jnrd3v

Methodology

Replicates

A single replicate with a matching input control used for all transcription factors/histone marks.

Sequencing depth

Library, Total Number of Reads, Uniquely-mapped Reads, Length of Reads, Paired/Single-end
CREBBP2_4B7_CutNRun, 114950513, 87009479, 50, Paired-end
CREBBP2_B5_CutNRun, 78576122, 59988151, 50, Paired-end
H3K27ac_4B7_ChIP, 39994438, 32529049, 50, Paired-end
H3K27ac_B5_ChIP, 42098199, 34689435, 50, Paired-end
Input_4B7_ChIP, 36508134, 28622238, 50, Paired-end
Input_4B7_CutNRun, 65345035, 46880652, 50, Paired-end
Input_B5_ChIP, 36820444, 28922889, 50, Paired-end
Input_B5_CutNRun, 63777519, 45391338, 50, Paired-end

Antibodies

CREBBP (A22)(Santa Cruz biotechnology, SC369, Lot: G1613) 1µg for CUTandRUN.
IgG (Proteintech, 30000-0-AP), 2.5µg
H3 (1B1B2) (Cell Signalling Technology, 14269S), 2.5µg
H3K27ac (Active motif, 39133) 2.5µg
IgG (Epicypher, cat 13-0042), 1µg

Peak calling parameters

MACS2 (version 2.2.7.1) was used to call peaks with the callpeak function and the following parameters (-p 0.05 -B --SPMR --keep-dup all).

Data quality

Black-listed regions were removed from the called peaks using bedops (version 2.4.41) and a list of genome- specific blacklisted region list from the human genome. Peaks with a minimum q-value of 0.05 were utilized for all downstream analysis.

Software

For ChIP-seq: FastQC, trimmomatic (version 0.36), Bowtie (version 2.4.5), Samtools (version 1.11), deeptools (version 3.5.1), MACS2 (version 2.2.7.1), bedops (version 2.4.41). For Cut&Run: CUT-RUNTools-2.0

Flow Cytometry

Plots

Confirm that:

[x] The axis labels state the marker and fluorochrome used (e.g. CD4-FITC).

[x] The axis scales are clearly visible. Include numbers along axes only for bottom left plot of group (a 'group' is an analysis of identical markers).

[x] All plots are contour plots with outliers or pseudocolor plots.

[x] A numerical value for number of cells or percentage (with statistics) is provided.

Methodology

Sample preparation

During flow cytometry cells were harvested by centrifugation at 300g for 5 minutes and washed with MACS buffer (DPBS, 2% HI-FBS and 2mM EDTA). Cell pellets were stained with the corresponding dye according to manufacturer's instructions: Annexin V (eBioscienceTM, 88-8007-74), MitoProbeTM JC-1 (ThermoFisher, M34152) and BODIPYC11 581/591 (ThermoFisher, D3861).
After incubation cells were centrifuged and resuspended in the corresponding viability dye or MACS buffer. DAPI (BD, 564907) was used for viability marker in the JC-1 experiments, whilst 7AAD (BD, 559925) was used with Annexin V.
The lentivirally transfected cells with shBCL2 or shRenilla express the reporter GFP or mCherry respectively. These cells were stained with viability marker DAPI.
The lentivirally transfected cells with FUCCI reporter system express the reporters mCherry and mVenus. These cells were stained with viability marker DAPI before flow cytometry and sorting.
For viability studies, 7AAD exclusion was measured.

Instrument

FACS cell sorting (BD Influx; BD Biosciences)
Flow cytometer (BD Fortessa; BD Biosciences)

Software

BD FACSDiva (8.0.1), FlowJo (10)

Cell population abundance

The purity of the double positive FUCCI cells was confirmed above 90% immediately post sort.

Gating strategy

FACS FUCCI double positive cell purification
1. Exclude debris (FSC / SSC)
2. Exclude doublets (FSC-H / FSC-A)
3. Gate viable cells (DAPI)
4. Gate and sort double positive cells for m-Venus and m-Cherry

Cell cycle analysis by FUCCI Reporter
1. Exclude debris (FSC / SSC)
2. Exclude doublets (FSC-H / FSC-A)
3. Gate viable cells (DAPI)
4. Gate (see Extended Figure 3e):
a) m-Cherry pos, m-Venus neg for G1
b) m-Cherry neg, m-Venus pos for G2-S-M
c) m-Cherry pos, m-Venus pos for Early S

Annexin V staining
1. Exclude debris (Broader gating FSC / SSC)
2. Exclude doublets (FSC-H / FSC-A)
3. Just for shRNA cells, a mCherry gating was performed.
4. Gate (see Figure 1g)
a) Annexin V APC pos and 7AAD neg cells for early apoptotic cells

JC-1
1. Exclude debris (FSC / SSC)
2. Exclude doublets (FSC-H / FSC-A)
3. Gate viable cells (DAPI)
4. Histogram of JC-1 (488nm 530/30)

Lipid peroxidation assays
1. Exclude debris (Stringent FSC / SSC)
2. Exclude doublets (FSC-H / FSC-A)
3. Histogram of FL1 (488nm 530/30) and FL3 (488nm 610/20)

☒ Tick this box to confirm that a figure exemplifying the gating strategy is provided in the Supplementary Information.
